# PhyloScan: identification of transcription factor binding sites using cross-species evidence

**DOI:** 10.1186/1748-7188-2-1

**Published:** 2007-01-23

**Authors:** C Steven Carmack, Lee Ann McCue, Lee A Newberg, Charles E Lawrence

**Affiliations:** 1 The Wadsworth Center, New York State Department of Health, Albany, NY 12201, USA; 2 Pacific Northwest National Laboratory, Richland, WA 99352, USA; 3 Departrnent of Computer Science, Rensselaer Polytechnic Institute, Troy, NY 12180, USA; 4 Division of Applied Mathematics, Brown University, Providence, RI 02912, USA

## Abstract

**Background:**

When transcription factor binding sites are known for a particular transcription factor, it is possible to construct a motif model that can be used to scan sequences for additional sites. However, few statistically significant sites are revealed when a transcription factor binding site motif model is used to scan a genome-scale database.

**Methods:**

We have developed a scanning algorithm, PhyloScan, which combines evidence from matching sites found in orthologous data from several related species with evidence from multiple sites within an intergenic region, to better detect regulons. The orthologous sequence data may be multiply aligned, unaligned, or a combination of aligned and unaligned. In aligned data, PhyloScan statistically accounts for the phylogenetic dependence of the species contributing data to the alignment and, in unaligned data, the evidence for sites is combined assuming phylogenetic independence of the species. The statistical significance of the gene predictions is calculated directly, without employing training sets.

**Results:**

In a test of our methodology on synthetic data modeled on seven *Enterobacteriales*, four *Vibrionales*, and three *Pasteurellales *species, PhyloScan produces better sensitivity and specificity than MONKEY, an advanced scanning approach that also searches a genome for transcription factor binding sites using phylogenetic information. The application of the algorithm to real sequence data from seven *Enterobacteriales *species identifies novel Crp and PurR transcription factor binding sites, thus providing several new potential sites for these transcription factors. These sites enable targeted experimental validation and thus further delineation of the Crp and PurR regulons in *E. coli*.

**Conclusion:**

Better sensitivity and specificity can be achieved through a combination of (1) using mixed alignable and non-alignable sequence data and (2) combining evidence from multiple sites within an intergenic region.

## Background

Alteration of the frequency of transcription from DNA to messenger RNA is the primary means by which an organism controls gene expression. Transcription initiation is controlled primarily through the binding of transcription factors (proteins) to cognate sites on a chromosome (transcription factor binding sites). For a given transcription factor and an experimentally identified set of transcription factor binding sites, or a set of co-regulated promoters, computational methods can be applied to identify the DNA sequence pattern that is recognized by the transcription factor. Such a sequence pattern is commonly referred to as a motif, which is a conceptual extension of a single sequence, in which each position is characterized not by a single nucleotide, but rather by a column vector representing the probability with which each of the four nucleotides contributes to the pattern at that position.

The prediction of additional transcription factor binding sites by comparison of a motif to the promoter regions of an entire genome is a vexing problem, due to the large database size (approximately one half million intergenic base pairs for a typical prokaryote, and several hundred million base pairs for a mammal) and the relatively small width of a typical transcription factor binding site (6–30 bp). In such a large search space, chance alone results in the identification of many sites that match the motif. The problem is further compounded by variability among the transcription factor binding sites that are recognized by a transcription factor; such variability permits differences in the level of regulation, due to the altered intrinsic affinities for the transcription factor [1].

Programs that use a motif to search (*i.e*., scan) a sequence database for matches (*i.e*., predicted transcription factor binding sites) fall into two general categories. One approach is to employ a training set of transcription factor binding sites and a scoring scheme to evaluate predictions [2-8]. The scoring scheme is often based on information theory [9], and the training set is used to empirically determine a score threshold for reporting of the predicted transcription factor binding sites. The second method relies on a rigorous statistical analysis of the predictions, based upon modeled assumptions. Briefly, the statistical significance of a sequence match to a motif can be assessed through the determination of type I error (*p*-value): the probability of observing a match with a score as good or better in a randomly generated search space of identical size and nucleotide composition. The smaller the *p*-value, the less likely that the match is due to chance alone. Staden [10] presented an efficient method that exactly calculates this probability, and Neuwald *et al*. [11] described an implementation of this method.

When either of the two types of method is used to scan an entire genome, or the promoter regions of a genome, there is a difficult trade-off between sensitivity and specificity. If the threshold for a prediction (sites above a chosen information measure cutoff, or below a chosen *p*-value level) is chosen so as to reflect a reasonably low false positive rate (*i.e*., high specificity), it is frequently difficult to recover many of the known transcription factor binding sites that were used in the construction of the motif. Conversely, the choice of a threshold for prediction that finds many of the known transcription factor binding sites (*i.e*., high sensitivity) invariably leads to an overwhelming number of additional predicted sites, most of which are likely false positives. (Generally, we do not know where a transcription factor might bind in a way that does not affect transcription and thus, in this latter case, the functional interpretation of these "false positives" is somewhat subtle.)

The goal of the present study has been to increase the statistical power, when scanning a genome sequence database with a regulatory motif, by taking advantage of additional sequence data from related species and from multiple sites within an intergenic region. We have extended Staden's method [10] to allow scanning of orthologous sequence data that are either multiply aligned, unaligned, or a combination of aligned and unaligned. Our new algorithm, PhyloScan, an extension of Staden's method, statistically accounts for the phylogenetic dependence of the species contributing data to the alignment and calculates a *p*-value for the sequence match in the aligned data set. This approach is similar to the MONKEY method [12]; however, there are several key differences between the two.

MONKEY requires that all sequences be multiply aligned. However, this requirement is too restrictive for many transcription factors of interest that are conserved across a broad phylogenetic range. That is, there are many cases in which distantly related species contain orthologous transcription factors and binding sites, even though general sequence alignments are not feasible (*e.g*., between eubacteria and archaea [13-15]). Thus, we have developed a scanning approach that will find sites in mixed data that can include one or more clades of sequences (each of which can be aligned reliably) as well as sequences which cannot be aligned reliably to any other sequences.

Furthermore, regulatory modules often include multiple sites, none of which alone would be statistically significant in a genome-scale scan. Our procedure addresses this important case. In addition, our procedure permits use of a wide range of nucleotide substitution models, and it reports *q*-values [16], the fraction of intergenic regions of a given strength or better that are expected to be false, whereas MONKEY reports *p*-values, the fraction of false sites expected to show a given strength or better.

## Results

We evaluated PhyloScan on both real and synthetic data. For the real data, we chose the *Escherichia coli *Crp and PurR motifs, and we gathered genome sequence data for several gamma-proteobacteria. We and others have previously demonstrated that a comparative genomic approach is effective in the prediction of transcription factor binding sites within this phylogenetic group [17-26]. Among the species chosen for this study (*E. coli, Salmonella enterica *serovar Typhi (*S. typhi*), *Yersinia pestis, Haemophilus influenzae, Vibrio cholerae, Shewanella oneidensis*, and *Pseudomonas aeruginosa*), only *E. coli *and *S. typhi *exhibit sufficient homology in the promoter regions [26]. Thus, we aligned orthologous intergenic regions for these two species, and we combined the statistical evidence from the scanning of the aligned *E. coli *and *S. typhi *data with the statistical evidence from the scanning of unaligned orthologous intergenic regions from the remaining five, more distantly related, species. (Approaches in which the *S. typhi *sequence data is considered independent of the *E. coli *sequence data were considered in earlier work [26].)

### Synthetic sequence data

While of interest for comparison with previous studies, this set of species is not representative of the problem of incorporating phylogeny into scanning methods. Furthermore, evaluation of scanning algorithms using real sequence data is difficult, because of the presence of transcription factor binding sites that are likely real, but unreported. That is, because they have not yet been experimentally verified, some predicted sites reported as false positives may, in fact, be true positives. Thus, we generated synthetic data in which we controlled the binding site content. Specifically, as a typical example, we generated four sets of sequence data modeled on the phylogenetic relationship of fourteen prokaryotic species: seven *Enterobacteriales *(*E. coli, S. typhi, Klebsiella pneumoniae, Salmonella bongori, Citrobacter rodentium, Shigella flexneri, & Proteus mirabilis*), four *Vibrionales *(*Vibrio cholerae, Vibrio parahaemolyticus, Vibrio vulnificus, & Vibrio fischeri*), and three *Pasteurellales *(*Haemophilus influenzae, Haemophilus somnus, & Haemophilus ducreyi*).

**Figure 1:**
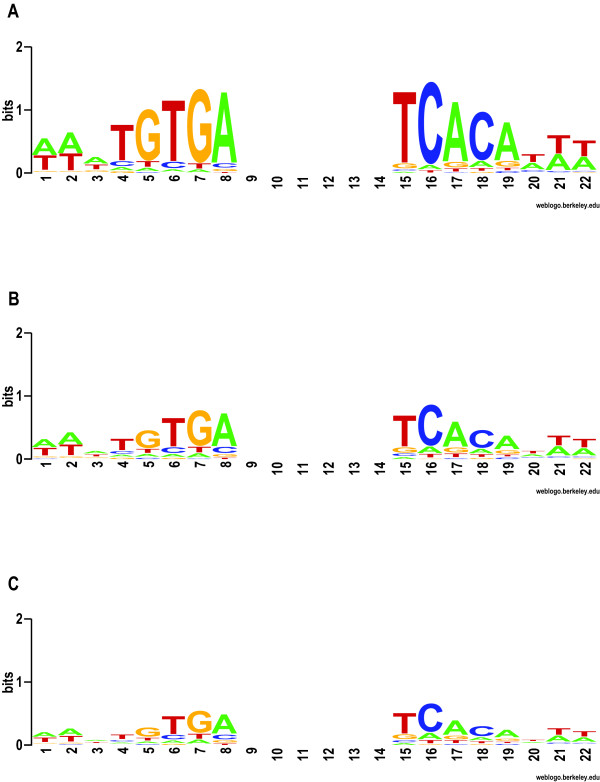
**Crp Binding Site Motif and Generation of Weaker Versions**. The logo in panel A indicates the Crp motif used to scan for Crp binding sites. It is also used to generate a pair of full-strength Crp sites in the synthetic sequence data. The binding site equilibria were calculated from sequence data aligned by the Gibbs Recursive Sampler [49], and were plotted using publicly available software [27]. The logo in panel B indicates the motif used to generate 1/2-strength Crp sites. It was generated by raising each probability of a nucleotide to its 0.637^th ^power, with subsequent scaling so that the probabilities of the four nucleotides for any motif column sum to 1.0. The exponent was chosen so that the average information content (*i.e*., "bits") would be half that value for the full-strength sites. The logo in panel C is the 1/3-strength Crp motif, generated with an exponent of 0.507 so that average information content would be one-third of the full-strength value.

The first synthetic data set consists of 140,000 simulated intergenic regions representing the orthologous promoter regions of 10,000 genes from the fourteen species, where each sequence is of length 500 bp, with two planted Crp sites, generated from the Crp motif model (Figure 1A). The second data set is the same but with "1/2-strength Crp" sites, where the average number of bits of information across the positions of a Crp motif is cut in half. The third data set contains "1/3-strength Crp" sites. The fourth data set is a negative control and contains no planted transcription factor binding sites. See the Methods and Figure 1 for more information.

**Figure 2:**
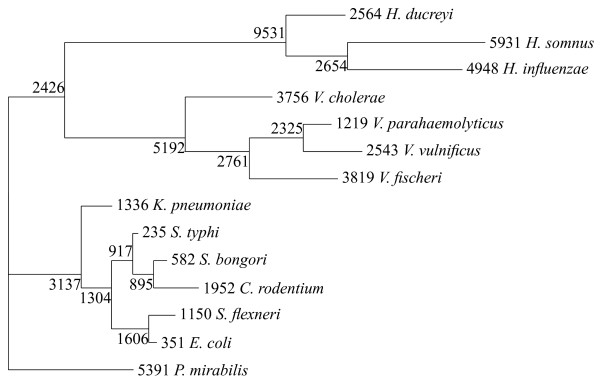
**Phylogenetic Tree of Fourteen Prokaryotes**. This tree of fourteen prokaryotes specifies the phylogenetic relationship of the species in our simulated sequence data. The tree is realistic, but approximate. The branch lengths represent the number of substitutions (including subsequent substitutions at a given sequence position) expected for each 10,000 nucleotides not subject to selection pressures.

With each simulated gene, the sequences were generated respecting the phylogenetic tree shown in Figure 2, using the nucleotide evolution model of Halpern & Bruno (1998) [28] for transcription factor binding sites and the model of Kimura (1980) [29] (with a transition to transversion ratio of 3.0) for background positions, and without the introduction of sequence gaps. The phylogenetic tree was generated from aligned (using MUSCLE [30]) 16S rRNA gene data via PHYLIP [31] and tree branch lengths were scaled up by a factor of 13.5 so that the tree would represent evolution at neutral sequence positions rather than at the somewhat conserved 16S rRNA gene sequence positions. Although the factor of 13.5 reflects our previous experience (unpublished), it is not rigorously chosen; for this and other reasons, although this tree is realistic, it should not be considered definitive.

Based upon the distances in the phylogenetic tree we partitioned the fourteen species into four clades, the *Vibrionales *clade, the *Pasteurellales *clade, *P. mirabilis *(by itself), and the remaining *Enterobacteriales *(henceforth, the *Enterobacteriales *clade). To evaluate the trade-off between sensitivity and specificity, we ran PhyloScan using the full-strength Crp motif; we scanned the full-strength-Crp-sites sequence data (positive data) and the no-sites sequence data (negative data). Likewise, we ran PhyloScan using the 1/2-strength Crp motif, scanning the 1/2-strength sequence data (positive data) and the no-sites sequence data (negative data); we also ran PhyloScan using the 1/3-strength Crp motif, scanning the 1/3-strength sequence data (positive data) and the no-sites sequence data (negative data).

Additionally, we ran PhyloScan with some of its features disabled. In three pairs of runs, one for each motif strength, as above, we ran PhyloScan on the four clades of sequence data, but by disabling its Neuwald-Green calculation (see Methods) we did not permit PhyloScan to statistically incorporate any sites other than the best found binding site in each intergenic region. In another three pairs of runs we ran PhyloScan, permitting it to consider multiple sites within an intergenic region, but by disabling its Bailey-Gribskov calculation (see Methods) PhyloScan could not consider more than one clade, and we gave it only the sequence data from the *Enterobacteriales *clade. Finally, we ran MONKEY (which incorporates neither the Neuwald-Green nor the Bailey-Gribskov calculation) on the *Enterobacteriales *clade sequence data, in a final three pairs of runs.

Each of these twelve pairs of runs – four algorithms times three motif strengths – produced *p*-values for each of 10,000 synthetic orthologous intergenic regions with sites and for each of 10,000 synthetic orthologous intergenic regions without sites. When any of the algorithms is used, it is desirable to set a *p*-value cutoff so that, in the positive data, the number of intergenic regions that have values below this cutoff is large and, in the negative data, the number of the intergenic regions that have values below the cutoff is small. Because the relative importances of the former (sensitivity) and the latter (type I error) depend upon the particular experiment and the parameters of that experiment, it is common to plot a Receiver Operating Characteristic (ROC) curve of sensitivity vs. type I error, to show what is achievable from differing cutoff levels.

**Figure 3:**
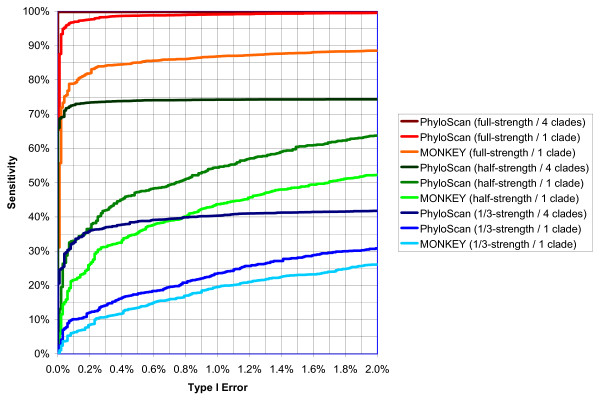
**ROC Curves for PhyloScan and MONKEY**. Shown are Receiver Operating Characteristic (ROC) curves for algorithms applied to intergenic regions containing a pair of full-strength Crp sites, a pair of 1/2-strength sites, and a pair of 1/3-strength sites. The simulated sequence data is for fourteen prokaryotic species organized into four clades; the orthologous intergenic sequences are 500 bp and are multiply-aligned within each clade but not between clades. ROC curves are shown for fully enabled PhyloScan and MONKEY. Additionally, ROC curves for PhyloScan applied to only the *Enterobacteriales *clade are shown. The ROC curves for PhyloScan with its multiple-clades capability enabled but its multiple-sites capability disabled are not shown because they are nearly indistinguishable from the fully enabled PhyloScan. A comparison of the "PhyloScan (1 clade)" curves to the "MONKEY (1 clade)" curves shows that there is value in combining evidence from multiple sites within an intergenic region using the Neuwald-Green calculation. A comparison of the "PhyloScan (4 clades)" curves to the "PhyloScan (1 clade)" curves indicates that there is additional value in considering data from multiple clades. For instance, if *p*-value cutoffs are chosen so that type I error is 0.1% (*i.e*., the specificity is 99.9%) then PhyloScan correctly classifies 99.85% of the full-strength-Crp intergenic regions, 72.68% of the 1/2-strength regions, and 32.64% of the 1/3-strength regions. The corresponding numbers for "PhyloScan (1 clade)" are 96.98%, 33.01%, and 10.11%. The corresponding numbers for MONKEY are 79.02%, 21.66%, and 6.33%. It is possible that sensitivities for the four-clades curves would have been even stronger if we had not prohibited the non-*Enterobacteriales *clades from rescuing intergenic regions in the *Enterobacteriales *clade that had failed to pass our 0.05 *p*-value cutoff.

Figure 3 shows the ROC curves for nine of the twelve cases; for our synthetic sequence data, the disabling of the Neuwald-Green calculation had negligible effect, and these three ROC curves are omitted. In all cases the disabling of both the Neuwald-Green and Bailey-Gribskov calculations significantly affected performance. (See Figure 3 and its legend for more information.)

### Real sequence data

To evaluate the statistical power provided by different facets of the PhyloScan approach in real sequence data, we measured the increase in sensitivity originating from three sources: a reduction in database size, the use of aligned sequence data only, and the use of non-alignable ortholog data.

As a stripped-down baseline, we applied PhyloScan in a scan of the full *E. coli *sequence database, ignoring all other sequence data; this baseline is equivalent to the original Staden method, and thus has the same statistical power.

We compared the baseline to the results achievable from a reduced database. When orthologous sequences are aligned between closely related species, gaps may be introduced, and there are often portions of the sequence that do not align; thus, the overall feasible search space for transcription factor binding sites is reduced. A search of such a reduced database in and of itself will allow the detection of more statistically significant transcription factor binding sites than will a search of a full set of intergenic regions from a single species. Therefore, the scanning results from a database reduced in size, yet containing data from only one species, will provide a measure of the increase in sensitivity to the baseline scan that is due simply to a reduction in search space.

We compared the baseline and reduced-database results to those obtained by scanning a database of aligned *E. coli-S. typhi *sequences, in order to measure the increase in sensitivity provided by the use of this aligned sequence data.

**Figure 4:**
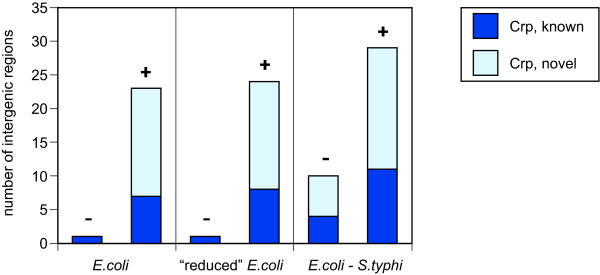
**Crp-Significant Intergenic Regions Found**. When counting Crp-significant intergenic regions, comparison of the bars labeled "+" (with the unalignable sequences) relative to those labeled "-" (without the unalignable sequences) indicates that the largest gain in sensitivity comes from the use of unalignable, evolutionarily distant sequences. The left part of this figure shows the sensitivity for the scan of *E. coli *data only. The center part of this figure shows the sensitivity from the scan of only those *E. coli *sequence data that are alignable with *S. typhi*. The right part of this figure shows the sensitivity from the scan of *E. coli-S. typhi *aligned sequence data.

**Figure 5:**
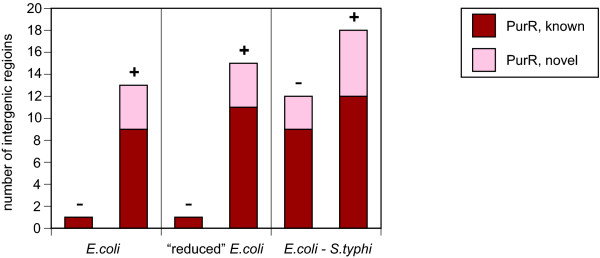
**PurR-Significant Intergenic Regions Found**. The results for PurR are similar to those for Crp. See the caption of Figure 4.

To test these sources of statistical power, we generated databases of promoter-containing *E. coli *intergenic regions, aligned *E. coli-S. typhi *intergenic regions, and motif models based on known Crp and PurR sites (see Methods). Specifically, the three databases contained: (1) the set of all *E. coli *intergenic regions, (2) the *E. coli *sequences extracted from the alignments of *E. coli-S. typhi *orthologous intergenic regions, and (3) the *E. coli-S. typhi *aligned intergenic regions data. Relative to the original method of Staden, our results show large improvement in the number of predicted transcription factor binding sites due to the alignment of two somewhat closely related species (Table 1 and Figures 4 and 5). Specifically, with a *q*-value cutoff of 0.001 (see Methods) the scanning of the set of all *E. coli *intergenic sequences results in only one Crp-significant intergenic region (with two predicted Crp sites), and one PurR-significant intergenic region (with one PurR site). No improvement was obtained in the reduced database of *E. coli *intergenic sequences. However, when the set of *E. coli-S. typhi *aligned sequences was scanned, 10 Crp-significant intergenic regions (with 13 Crp sites total), and 12 PurR-significant intergenic regions (with 13 PurR sites total) were predicted.

**Table 1:** Summary of PhyloScan Predictions

	C1	C2	C3	C4	C5	C6
*E. coli *Sequence Data	Full^*a*^	Full^*a*^	Red.^*b*^	Red.^*b*^	Red. & Aligned^*c*^	Red. & Aligned^*c*^

Indep. Species	No	Yes	No	Yes	No	Yes

Crp Known^*d*^	1(2)	7(10)	1(2)	8(12)	4(6)	11(16)
Crp Novel^*d*^	0(0)	16(20)	0(0)	16(18)	6(7)	18(21)
PurR Known^*d*^	1(1)	9(9)	1(1)	11(11)	9(9)	12(12)
PurR Novel^*d*^	0(0)	4(5)	0(0)	4(5)	3(4)	6(7)

This table shows the number of *E. coli *intergenic regions predicted by PhyloScan to contain Crp or PurR binding sites, with the total number of sites predicted within parentheses. Column C1 is for a scan of the full set of *E. coli *intergenic sequence data (excluding the *S. typhi *sequence data and the sequence data from the other, independent clades). Column C3 is for a scan of only that *E. coli *sequence that is alignable with *S. typhi*; the *S. typhi *sequence data continue to be excluded. Column C5 is for a scan of the aligned *E. coli-S. typhi *sequence data. Columns C2, C4, and C6, are like Columns C1, C3, and C5, respectively, but the sequence data from the independent clades are also incorporated. Observing the lack of improvement of Column C3 over Column C1 (or the meager improvement of C4 over C2), we conclude that there is minimal gain in sensitivity from considering only *E. coli *sequence that is alignable with *S. typhi*, when not actually using the aligned *S. typhi *sequence data. Observing the modest improvement of C5 over C3 (or C6 over C4), we conclude that incorporating the aligned *S. typhi *sequence gives a moderate gain in sensitivity. Observing the large improvement of C2 over C1 (or C4 over C3, or C6 over C5), we conclude that incorporating the data from species that are not alignable with *E. coli *gives a significant gain in sensitivity. Notes: ^*a*^Database of 2379 intergenic sequences from *E. coli *[see Additional file 2]. ^*b*^Database of *E. coli *sequences (reduced search space) extracted from the *E. coli-S. typhi *database (see Real Sequence Data in Results). ^*c*^Database of *E. coli-S. typhi *aligned intergenic sequences (see Real Sequence Data in Results). ^*d*^The number of *E. coli *intergenic regions predicted by PhyloScan to contain Crp or PurR binding sites, where the total number of binding sites detected is in parentheses and those sites that correspond to known, experimentally verified transcription factor binding sites and those sites that are novel (not yet verified) are indicated.

Furthermore, in each of the tests described above (using the baseline, the reduced-database, or the aligned sequence data) we can incorporate non-alignable orthologous sequence data to measure the impact of these additional data on sensitivity. Thus, to determine the extent to which additional, more distantly related, species could provide evidence to support a particular candidate transcription factor binding site upstream of a particular gene in the target species, we used PhyloScan to scan the orthologous intergenic regions for that candidate gene from the additional species (clades), assuming phylogenetic independence between clades. The *p*-value representing the combined evidence supporting a transcription factor binding site prediction was then calculated using the method of Bailey and Gribskov [32], as described in the Methods.

To demonstrate this approach with the *E. coli *Crp and PurR examples, we employed orthologous data from the five additional gamma-proteobacterial species listed above. We used PhyloScan to identify potential Crp and PurR transcription factor binding sites in the *E. coli*-only and *E. coli-S. typhi *aligned data sets, using a *P*_intergenic _≤ 0.05 cutoff to select candidate intergenic regions for examination in the other five species. As summarized in Table 1, depicted in Figures 4 and 5, and described below, we observed a considerable increase in the number of predicted transcription factor binding sites at the *q*-value ≤ 0.001 level, when the evidence from the five additional gamma-proteobacterial species was included by combining *p*-values.

For example, PhyloScan identified a total of 10 Crp-significant intergenic regions in the *E. coli-S. typhi *aligned data, but after combination of the evidence from the remaining five species, a total of 29 Crp-significant intergenic regions were predicted, a near tripling. Compared to a simple search of the raw *E. coli *intergenic sequences (one Crp-significant intergenic region), this represents a tremendous increase in sensitivity. The results with the PurR model were also dramatic: the use of data from *S. typhi, Y. pestis, H. influenzae*, and *V. cholerae *provided a 50% increase in the number of PurR-significant intergenic regions (to 18 from 12), compared to the scanning of *E. coli-S. typhi *aligned intergenic sequences only. In the *E. coli *sequence alone there was only a single PurR-significant intergenic region. In the Supplementary Materials are tables listing the located sites for Crp [see Additional file 3] and PurR [see Additional file 4], as well as captions for these tables [see Additional file 1].

We also examined the best 20 reported intergenic regions for each of the six approaches shown in Table 1. We see several differences, not only in the reported *q*-values, but also in the order and appearance of predicted binding sites in intergenic regions; see the caption of Table 2 for more details.

**Table 2:** Top 20 Predictions by PhyloScan

	C1		C2		C3		C4		C5		C6	
*E. coli *Sequence	Full^*a*^		Full^*a*^		Reduced^*b*^		Reduced^*b*^		Reduced & Aligned^*c*^		Reduced & Aligned^*c*^	

Indep. Species	No		Yes		No		Yes		No		Yes	

Rank	Gene	log(*q*)	Gene	log(*q*)	Gene	log(*q*)	Gene	log(*q*)	Gene	log(*q*)	Gene	log(*q*)

1	yibI	-4.65	cdd	-9.28	mtlA	-5.14	mtlA	-9.76	mtlA	-7.66	mtlA	-12.15
2	yqcE	-2.86	glpT	-7.21	ygcW	-2.89	cdd	-9.60	yjcB	-4.55	glpA	-9.19
3	b1904	-2.61	mglB	-6.01	yjcB	-2.62	glpA	-8.31	gcd	-3.99	cdd	-9.16
4	fucA	-2.51	yibI	-5.26	yjiY	-2.60	mglB	-6.53	b2146	-3.97	mglB	-7.60
5	deaD	-2.51	yjiY	-4.57	b2146	-2.53	gapA	-5.21	fucA	-3.93	udp	-6.26

6	yjiY	-2.42	hemC	-4.38	fucA	-2.51	udp	-5.17	ygcW	-3.42	gapA	-6.02
7	cdd	-2.29	deaD	-4.35	deaD	-2.47	yjiY	-4.79	flhD	-3.03	yjcB	-5.09
8	yeaA	-2.22	ysgA	-4.33	cdd	-2.31	cyaA	-4.70	gapA	-3.03	cyaA	-5.04
9	yhcR	-2.06	yhcR	-3.99	gapA	-2.22	deaD	-4.37	ycdZ	-3.01	malE	-4.83
10	ycdZ	-1.96	yqcE	-3.56	qseA	-2.03	malE	-4.29	udp	-2.78	ycdZ	-4.69

11	b2736	-1.87	adhE	-3.47	ycdZ	-1.98	ygcW	-3.63	b2248	-2.76	adhE	-4.56
12	uxaC	-1.81	ycdZ	-3.45	mglB	-1.90	adhE	-3.58	glpA	-2.76	b2146	-4.53
13	ysgA	-1.77	yeaA	-3.44	udp	-1.86	ycdZ	-3.52	mglB	-2.73	fucA	-4.46
14	glpT	-1.75	mlc	-3.37	uxaC	-1.85	mlc	-3.48	qseA	-2.68	pckA	-4.09
15	mglB	-1.63	b1904	-3.31	glpA	-1.84	fucA	-3.32	pckA	-2.36	aer	-3.97

16	pckA	-1.39	fucA	-3.23	pckA	-1.45	yjcB	-3.32	adhE	-2.14	ygcW	-3.78
17	serA	-1.23	b2736	-3.18	malE	-1.36	pckA	-3.23	aer	-2.13	gcd	-3.67
18	aer	-1.23	pckA	-3.17	aer	-1.32	aer	-3.17	cdd	-2.10	deaD	-3.65
19	adhE	-1.22	aer	-3.08	serA	-1.32	qseA	-3.07	deaD	-2.04	serA	-3.62
20	mlc	-1.01	yjeG	-3.05	adhE	-1.28	uxaC	-3.07	uxaC	-2.02	mlc	-3.62

# Diffs from C6		10		11		3		3		4		0

Because it is sometimes instructive to examine a fixed number of top hits regardless of the reported *q*-values, in this table we compare the six approaches' best 20 intergenic regions for Crp. By comparing each column to Column C6, which is the best approach we employed, we see that the C1-C5 approaches give significantly different *q*-values for, and orderings of, the predicted regulated genes. As indicated in the bottom row, the C1-C5 approaches miss several of the top-20 genes reported in C6, replacing them with genes that did not make the C6 top-20 list. In particular, although it uses all of the sequence data except *S. typhi*, C2 is significantly different from C6. Furthermore, although C3 has few differences from C6 in the set of genes indicated, the *q*-values of C3 are considerably worse and the gene order is substantially rearranged. These data suggest that the ability to simultaneously handle both aligned and unaligned data is important in obtaining accurate predictions. Notes: ^*abc*^See the caption notes for Table 1. Also see the Table 1 caption for descriptions of Columns C1-C6.

It is worth noting here that the non-alignable species were selected for combination of *p*-values based upon the presence or absence of the transcription factor under study. All gamma-proteobacteria used in this study encode orthologs to Crp; hence, data for all species were included when *p*-values were combined from scans with the Crp motif. In contrast, because *S. oneidensis *and *P. aeruginosa *do not encode PurR orthologs, these species were not considered when we scanned for PurR binding sites.

## Discussion

### Key features of PhyloScan

We are able to increase the flexibility and sensitivity of scanning, without increasing the false positive rate, by incorporating the following three key features into PhyloScan:

1. We allow a mixture of alignable and unalignable sequence data. Specifically, sequences that can be reliably multiply aligned should be grouped and aligned. These clades of multiply-aligned sequences, including each "degenerate clade" of one sequence that cannot be reliably aligned with any other sequence, are used by PhyloScan. A phylogenetic tree relating the sequences within a clade, a user-specified nucleotide substitution model, and an extension to Staden's precise *p*-value calculation that is phylogenetically aware are all employed by PhyloScan to increase the statistical power of Staden's original method. (See Methods.)

2. We combine evidence from multiple sites within an intergenic region to produce a better sensitivity than could be achieved by simply examining the strongest site within an intergenic region. Specifically, a group of weak sites, none of which is statistically significant in isolation, is detected by the fact that for some value *i*, the *i*th weakest of the sites is surprisingly strong given that it is the *i*th weakest. (See Methods.)

3. We report our findings in terms of *q*-values [16] instead of *p*-values. For each intergenic region we report the probability that a region of its significance or better will be a false prediction, instead of reporting the probability that a negative control will appear at this significance or better.

### Applicability of PhyloScan

The test cases described here reflect our past and present research interests in proteobacterial gene regulation, while simultaneously emphasizing PhyloScan's ability to handle multiple weak binding sites as well as mixed aligned and unaligned sequence data. However, the features of our data set are not unique; there are many examples where multiple binding sites are common (*e.g*., flies [33] and humans [34]) or where transcription factors and their cognate binding sites are conserved across diverse species for which multiple sequence alignments are not feasible (*e.g*., between eubacteria and archaea [13-15]). PhyloScan will have clear advantages in such contexts. However, it is important to note that in situations where orthologous regions are usually alignable and for which the multiple-weak-sites scenario is unlikely, PhyloScan will not perform better than existing approaches such as MONKEY. In another direction, in cases where sequences cannot be aligned, PhyloScan will not perform better than existing approaches that handle "independent species."

Here we have demonstrated significant improvement of scan results through the use of sequences from evolutionary distant species that have orthologous transcription factors. This is not unexpected, given results of a more theoretical nature that quantify the extent of such improvement [35].

### PhyloScan evaluates significance at the level of the intergenic region

A key focus of this work has been to combine evidence across transcription factor binding sites within an intergenic region and across orthologous regions in order to correctly identify intergenic regions that are likely to contain transcription factor binding sites, even when each of the identified transcription factor binding sites, considered in isolation, may not be sufficiently strong to be statistically significant. Accordingly, the individual sites included in our predictions are not necessarily statistically significant and individual site predictions may be false positives even within true-positive intergenic sequences.

For instance, in the collection of 10,000 synthetic data sets in which we planted two full-strength Crp transcription factor binding sites per intergenic region, we have 9,985 true positive intergenic regions at the 99.9% specificity level (see Figure 3). Of these true positives, in 6,287 of the *E. coli *intergenic regions two sites were predicted and the sites exactly coincided with the two planted sites. In 24 *E. coli *intergenic regions two sites were predicted and one of the two sites exactly coincided with a planted site. In 3,672 of these regions one site was predicted and it exactly coincided with one of the two planted sites, and in 2 of the *E. coli *intergenic regions, one site was predicted that did not exactly coincide with a planted site.

### Key user-selectable parameters in PhyloScan

#### Focus on a target species or clade

In running PhyloScan, the user must specify two cutoff values, and can optionally specify additional parameters describing the expected multiplicity of binding sites upstream of a regulated gene. The first cutoff is a *p*-value cutoff, calculated on a per intergenic-sequence basis for the clade that includes the species of primary interest. We chose a default value of 0.05, so that weak intergenic regions in the target species' clade will not be considered, even when strong intergenic regions are located in orthologous regions in more-distantly related species. The choice of a larger value would reduce the focus on the target species, allowing strong sites in other species to rescue weak sites in the target species. The choice of a smaller value would increase the focus on the target species; the choice of a very small value would effectively cancel out the information available from the related species, since any intergenic region that looks extremely promising in the target species will almost surely continue to look promising when additional data are included.

#### Quality of reported sites

The second cutoff that our approach requires is the *q*-value cutoff that specifies which sites will be reported. We chose a default value of 0.001, meaning that according to our model, at most 0.1% of the intergenic sequences that we report as binding the transcription factor are chance false positives. While we have incorporated a fairly accurate phylogenetic model, we have not incorporated into this model such effects as the non-independence of the positions in a site (*e.g*., the effect of di- or tri-nucleotide energy terms, also known as stacking energies), nor effects from the cooperative binding of multiple transcription factors on the ability of a factor to bind to a DNA site. Because our model does not capture these and other features, the actual rate of false positives is likely to be higher than 0.1%.

On the other hand, in calculating the *q*-value, we have assumed that the vast majority of intergenic sequences in a genome will likely not contain a transcription factor binding site for the particular transcription factor under study, *i.e*., we are looking for rare events. Under this assumption, the proportion of all intergenic sequences that are truly null will approach 1.0 in Storey and Tibshirani's *q*-value calculation (the π^0
 MathType@MTEF@5@5@+=feaafiart1ev1aaatCvAUfKttLearuWrP9MDH5MBPbIqV92AaeXatLxBI9gBaebbnrfifHhDYfgasaacH8akY=wiFfYdH8Gipec8Eeeu0xXdbba9frFj0=OqFfea0dXdd9vqai=hGuQ8kuc9pgc9s8qqaq=dirpe0xb9q8qiLsFr0=vr0=vr0dc8meaabaqaciaacaGaaeqabaqabeGadaaakeaaiiGacuWFapaCgaqcamaaBaaaleaacqaIWaamaeqaaaaa@2F9A@ term of [16]), and so does not appear in our *q*-value equation (see Methods). In a case where this assumption does not hold, the *q*-values provided by our approach will be overly conservative.

Note that the scan technology, first described by Staden [10] and employed here, is a frequentist hypothesis testing approach. A Bayesian approach presents an alternative through the use of Bayesian posterior probabilities for each site. Such an approach would require the specification of a model from which alternative sequences are drawn as well as null sequences. When a large number of observations are available the approach of Efron *et al*. [36] provides a compromise that yields local false discovery rates through the use of empirical Bayesian methods.

#### The number of sites per intergenic region

The number of potential sites to consider in each intergenic region, and their respective weights, are additional parameters that can be set by the user to best capture the underlying biology in the system under study. Generally speaking, for *i *≥ 1, the algorithm detects that an intergenic region with sites is significant when its *i*th best site is surprisingly strong given its rank as the *i*th best site. The weight *w*_*i *_should be chosen in proportion to the number of such intergenic regions that are expected to have *i *as the first/lowest rank that appears strong by this test. We have set the default to have weights (*w*_1_, *w*_2_) = (0.9, 0.1) under the assumption that approximately 90% of intergenic regions with sites will have a strong site; among the remaining intergenic regions with sites, nearly all will have a site that is surprisingly strong given its rank as second strongest. (See the Methods.)

### Divergently transcribed genes

The presence of divergently transcribed genes, that is, the circumstance in which an intergenic region is upstream of, and contains the promoters for, both of a given pair of neighboring genes, is quite common in prokaryotes, and also occurs in eukaryotes, albeit much less frequently. Divergently transcribed genes occur frequently in the *E. coli *genome (644 pairs of divergently transcribed genes), and their presence has raised the question of which orthologous data should be used when we combine *p*-values. In the present implementation of PhyloScan, the choice was made randomly. Thus, in such cases, we were as likely to make a "correct" choice as to make an "incorrect" choice, if only one of the *E. coli *genes flanking an intergenic region containing candidate transcription factor binding sites is regulated by the transcription factor of interest. However, in cases where gene synteny is conserved across several species, this choice becomes irrelevant. That is, when synteny is conserved, the same intergenic regions from each species will be examined regardless of the gene chosen; inspection of the output and, ultimately, experimental validation become necessary in order to evaluate whether a predicted site is associated with the chosen gene, with the divergently transcribed gene, or with both. Implementation of a systematic or informed choice in these situations will be a topic for the future development of PhyloScan.

## Conclusion

We have used PhyloScan to combine evidence from matching sites found in orthologous data from several related bacterial species. In simulated sequence data, we demonstrate good sensitivity at high specificity levels. In real sequence data we are able to rediscover many of the known Crp and PurR transcription factor binding sites in *E. coli*, and we predict several novel Crp-significant intergenic regions and several novel PurR-significant intergenic regions in *E. coli*; specifically, over half of the Crp sites and one-third of the PurR sites are not experimentally validated by DNase I or electrophoretic mobility shift assays. Accordingly, our results have provided several new potential binding sites for these transcription factors, that require validation, to enable further delineation of these regulons in *E. coli*.

Through its capability of using cross-species data, PhyloScan improves the sensitivity of motif scanning; because the approach permits the use of both aligned and unaligned data, from both evolutionarily near and somewhat more distant species, it is our hope that researchers will find it useful in a wide variety of settings.

PhyloScan is available on request from the authors via phyloscan@wadsworth.org, and a Web interface for the software is available [37].

## Methods

Like the MONKEY method [12], PhyloScan uses the phylogenetic model of Neyman [38] and the efficient algorithm of Felsenstein [39] to evaluate the probability that a site in observed multiply-aligned sequence data is consistent with a transcription factor's motif model. With either MONKEY or PhyloScan, each position of the motif is evaluated, and the computed probabilities for the motif positions are then multiplied together to give the strength of the site. Via the approach of Staden [10], the probability that such strength would arise by chance is precisely computed.

PhyloScan goes beyond MONKEY in several key ways. First, PhyloScan combines the information from multiple sites within an intergenic region, so that evidence from weak sites that would not be significant in isolation is combined, to identify a statistically significant find. Second, information from more-distant sequences, both non-alignable isolated sequences and clades of alignable sequences, is incorporated so as to further increase sensitivity, without an accompanying increase in false predictions. Third, we signify strength of a find by reporting its *q*-value, the fraction of predictions of this probability or better that are expected to be false, rather than its *p*-value, the fraction of false sites that are expected to demonstrate this probability or better.

Descriptions of the three main differences between the two algorithms are provided below.

### Combining evidence across sites within an intergenic region

PhyloScan combines information from multiple predictions via a weighted Bonferroni test in a manner similar to that of Neuwald and Green [40]. Specifically, for a user-supplied value *k*, which defaults to 2, and user-supplied weights (*w*_1_,..., *w*_*k*_), which default to (0.9, 0.1), PhyloScan conservatively computes an intergenic region's *p*-value as

Pintergenic=min⁡{1,p1w1,p2w2,...,pkwk}
 MathType@MTEF@5@5@+=feaafiart1ev1aaatCvAUfKttLearuWrP9MDH5MBPbIqV92AaeXatLxBI9gBaebbnrfifHhDYfgasaacH8akY=wiFfYdH8Gipec8Eeeu0xXdbba9frFj0=OqFfea0dXdd9vqai=hGuQ8kuc9pgc9s8qqaq=dirpe0xb9q8qiLsFr0=vr0=vr0dc8meaabaqaciaacaGaaeqabaqabeGadaaakeaacqWGqbaudaWgaaWcbaGaeeyAaKMaeeOBa4MaeeiDaqNaeeyzauMaeeOCaiNaee4zaCMaeeyzauMaeeOBa4MaeeyAaKMaee4yamgabeaakiabg2da9iGbc2gaTjabcMgaPjabc6gaUnaacmqabaGaeGymaeJaeiilaWYaaSaaaeaacqWGWbaCdaWgaaWcbaGaeGymaedabeaaaOqaaiabdEha3naaBaaaleaacqaIXaqmaeqaaaaakiabcYcaSmaalaaabaGaemiCaa3aaSbaaSqaaiabikdaYaqabaaakeaacqWG3bWDdaWgaaWcbaGaeGOmaidabeaaaaGccqGGSaalcqGGUaGlcqGGUaGlcqGGUaGlcqGGSaaldaWcaaqaaiabdchaWnaaBaaaleaacqWGRbWAaeqaaaGcbaGaem4DaC3aaSbaaSqaaiabdUgaRbqabaaaaaGccaGL7bGaayzFaaaaaa@5AAD@

where the weights (*w*_1_,..., *w*_*k*_) are nonnegative and sum to one, and *p*_*i *_is the probability that a randomly generated, intergenic sequence alignment of the same size would have its *i*th best site as good as or better than the *i*th best site in the intergenic sequence data under consideration. The calculation is conservative because the underlying events whose probabilities are (*p*_1_,..., *p*_*k*_) are not statistically disjoint [40].

Thus, an intergenic region with a strong site will make its presence known via a strong (*i.e*., low) value for the *p*_1_/*w*_1 _term, and an intergenic region that does not have a strong site, but that does have an *i*th best site that is surprisingly strong (given its rank as *i*th best), will be detected through a strong value for the *p*_*i*_/*w*_*i *_term. This enables us to detect both transcription factors that tend to bind strongly but in isolation and transcription factors that tend to bind multiply but weakly.

An alternate approach for combining the contributions of multiple binding sites, that of seeking the *p*-value of the sum of their log-likelihoods [41], is not employed by PhyloScan.

### Combining evidence from more-distant sequences

As described above, a *P*_intergenic  _*p*-value is generated for each sequence alignment of an intergenic region, but a true site's value may still be too weak to distinguish that site from the false positives in a vast genome. To address this problem, we combine this *p*-value with the *p*-values for the same intergenic region that come from sequence alignments of more distantly-related species. That is, we partition the input sequences for orthologous promoters into clades such that each clade is either an isolated sequence or contains sequences that can be reliably, multiply aligned; we compute the *P*_intergenic _value for each clade as above; and we combine these *p*-values using the formula of Bailey and Gribskov [32]. When there are *n *such clades whose *P*_intergenic _values are *P*_1_, *P*_2_,..., *P*_*n *_then we compute:

Pproduct=∏c=1nPcPcombined=Pproduct∑i=0n−1(−ln⁡(Pproduct))ii!.
 MathType@MTEF@5@5@+=feaafiart1ev1aaatCvAUfKttLearuWrP9MDH5MBPbIqV92AaeXatLxBI9gBaebbnrfifHhDYfgasaacH8akY=wiFfYdH8Gipec8Eeeu0xXdbba9frFj0=OqFfea0dXdd9vqai=hGuQ8kuc9pgc9s8qqaq=dirpe0xb9q8qiLsFr0=vr0=vr0dc8meaabaqaciaacaGaaeqabaqabeGadaaakeaafaqaaeGadaaabaGaemiuaa1aaSbaaSqaaiabbchaWjabbkhaYjabb+gaVjabbsgaKjabbwha1jabbogaJjabbsha0bqabaaakeaacqGH9aqpaeaadaqeWbqaaiabdcfaqnaaBaaaleaacqWGJbWyaeqaaaqaaiabdogaJjabg2da9iabigdaXaqaaiabd6gaUbqdcqGHpis1aaGcbaGaemiuaa1aaSbaaSqaaiabbogaJjabb+gaVjabb2gaTjabbkgaIjabbMgaPjabb6gaUjabbwgaLjabbsgaKbqabaaakeaacqGH9aqpaeaacqWGqbaudaWgaaWcbaGaeeiCaaNaeeOCaiNaee4Ba8MaeeizaqMaeeyDauNaee4yamMaeeiDaqhabeaakmaaqahabaWaaSaaaeaacqGGOaakcqGHsislcyGGSbaBcqGGUbGBcqGGOaakcqWGqbaudaWgaaWcbaGaeeiCaaNaeeOCaiNaee4Ba8MaeeizaqMaeeyDauNaee4yamMaeeiDaqhabeaakiabcMcaPiabcMcaPmaaCaaaleqabaGaemyAaKgaaaGcbaGaemyAaKMaeiyiaecaaiabc6caUaWcbaGaemyAaKMaeyypa0JaeGimaadabaGaemOBa4MaeyOeI0IaeGymaedaniabggHiLdaaaaaa@7A2C@

This formula precisely computes the *p*-value for the product of *n *values drawn randomly from the interval [0, 1]. An example of this calculation is available in the Supplementary Materials [see Additional file 1].

PhyloScan allows a *p*-value cutoff *α*, which defaults to 0.05, such that sites in a user-specified clade of interest that are worse than this cutoff are not permitted to be strengthened by data from the other species via the combination process. This feature allows the user to concentrate on a single clade or species rather than the entire tree of species. Because of this cutoff, it is appropriate to modify the above formula for sites that survive the cutoff:

Pcombined=Pproduct∑i=0n−1(−ln⁡(Pproductα))ii!.
 MathType@MTEF@5@5@+=feaafiart1ev1aaatCvAUfKttLearuWrP9MDH5MBPbIqV92AaeXatLxBI9gBaebbnrfifHhDYfgasaacH8akY=wiFfYdH8Gipec8Eeeu0xXdbba9frFj0=OqFfea0dXdd9vqai=hGuQ8kuc9pgc9s8qqaq=dirpe0xb9q8qiLsFr0=vr0=vr0dc8meaabaqaciaacaGaaeqabaqabeGadaaakeaafaqabeqadaaabaGaemiuaa1aaSbaaSqaaiabbogaJjabb+gaVjabb2gaTjabbkgaIjabbMgaPjabb6gaUjabbwgaLjabbsgaKbqabaaakeaacqGH9aqpaeaacqWGqbaudaWgaaWcbaGaeeiCaaNaeeOCaiNaee4Ba8MaeeizaqMaeeyDauNaee4yamMaeeiDaqhabeaakmaaqahabaWaaSaaaeaadaqadaqaaiabgkHiTiGbcYgaSjabc6gaUnaabmaabaWaaSaaaeaacqWGqbaudaWgaaWcbaGaeeiCaaNaeeOCaiNaee4Ba8MaeeizaqMaeeyDauNaee4yamMaeeiDaqhabeaaaOqaaGGaciab=f7aHbaaaiaawIcacaGLPaaaaiaawIcacaGLPaaadaahaaWcbeqaaiabdMgaPbaaaOqaaiabdMgaPjabcgcaHaaacqGGUaGlaSqaaiabdMgaPjabg2da9iabicdaWaqaaiabd6gaUjabgkHiTiabigdaXaqdcqGHris5aaaaaaa@65F7@

### Utility of *q*-value over *p*-value

The *p*-value, the probability that a negative control would appear positive, must be used with great care because genomes are vast relative to regulatory sequence elements. For instance, in many other situations a *p*-value of 10^-6 ^is considered excellent, but when there are on the order of 10^9 ^places where a transcription factor binding site is not likely to bind, such a "strong" *p*-value can leave us with 1,000 false positives – or even more, in the usual case that some of the biology has not been incorporated into the statistical model. Thus, to properly interpret a *p*-value, the researcher must be on guard to quantify the number of negative cases.

The *q*-value (or False Discovery Rate [16]) explicitly incorporates the vastness of the genome in the calculation. The *q*-value of a transcription factor binding site tells us the proportion of sites of that strength or better that we expect to be false positives. Under ideal circumstances, the researcher who chooses a *q*-value threshold of 0.001 expects only one in 1,000 of the reported sites to be a false positive regardless of the genome size. (However, because we do not pretend to have statistically modeled all the relevant biology, the false discovery rate will generally be higher than the specified threshold.)

### Real data inputs

**Figure 6:**
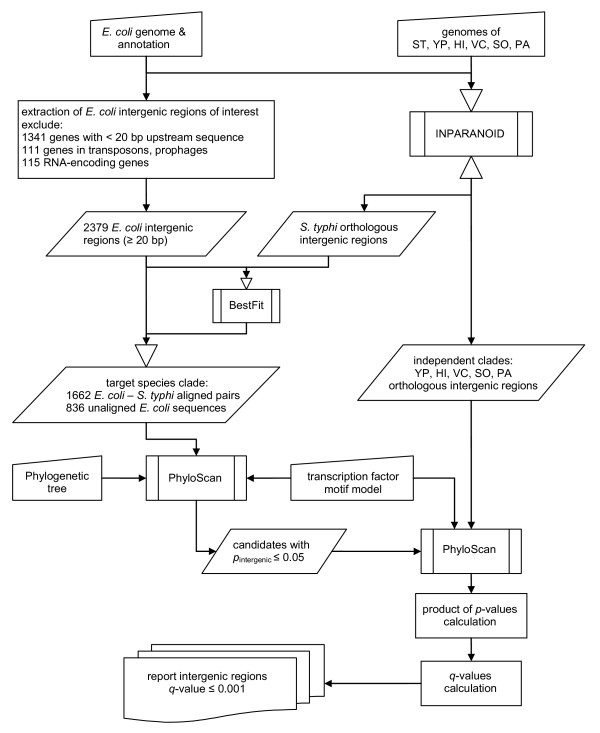
**Data Processing Flow Chart for PhyloScan**. An overview of the steps taken to locate Crp and PurR transcription factor binding sites in *E. coli *intergenic regions. The species examined were *Escherichia coli *(EC), *Salmonella enterica *serovar Typhi (*S. typhi*) (ST), *Yersinia pestis *(YP), *Haemophilus influenzae *(HI), *Vibrio cholerae *(VC), *Shewanella oneidensis *(SO), and *Pseudomonas aeruginosa *(PA).

The collection of orthologous intergenic regions, the division of species into clades, the multiple alignments, the phylogenetic trees, and the motif models needed as input to PhyloScan (or other similar algorithms) can be difficult to construct, and are unique to an individual's research interests and applications. We discuss our approaches in the following. The flowchart in Figure 6 depicts a high-level view of the intergenic sequence database generation and the application of PhyloScan to these data.

It is our belief that PhyloScan (and, *e.g*., MONKEY) are fairly robust to typical levels of error in these inputs, though further exploration is required to substantiate this claim.

#### Locating orthologous sequences

Genome sequence data and annotations were downloaded from the NCBI RefSeq database [42]: *Escherichia coli *K12 (NC_000913.1), *Salmonella enterica *serovar Typhi (*S. typhi*)(NC_003198), *Yersinia pestis *CO92 (NC_003143), *Haemophilus influenzae *Rd (NC_000907), *Vibrio cholerae *El Tor (NC_002505 and NC_002506), *Shewanella oneidensis *MR-1 (NC_004347 and NC_004349), and *Pseudomonas aeruginosa *PA01 (NC_002516). Orthologs for each of the annotated *E. coli *genes were identified in each of the remaining six species, using INPARANOID v.1.35 [43]. This program uses BLAST [44] to compare the complete set of predicted protein sequences from one genome with that of another, and identifies the reciprocal best hits. We set the parameters to use the BLOSUM62 matrix and a minimum bit score of 30, and we required that the alignment cover at least 50% of both proteins.

In the examples presented in this study, *E. coli *was the primary species of interest; we therefore identified a set of *E. coli *promoter-containing sequences by identifying each *E. coli *protein-coding gene (excluding 111 genes encoded on transposons or prophage elements) that has at least 20 bp of upstream intergenic sequence. By these criteria, there are 2379 *E. coli *intergenic regions of interest. Orthologous upstream intergenic-sequence data files were then generated for this set of 2379 *E. coli *regions, using the results from INPARANOID to identify orthologs, and the seven genome annotations to define intergenic boundaries. In the Supplementary Materials are a table with these data [see Additional file 2] and a caption for the table [see Additional file 1].

#### Designating clades

Among the species included in this study, only *E. coli *and *S. typhi *exhibit extensive homology (70% identity on average) in the promoter regions [26]. The phylogenetic distance of two sequences that share this level of homology is 0.384, assuming the nucleotide substitution model of Jukes & Cantor [45] (and the value would be similar under a variety of more current models); thus, we assumed this phylogenetic distance between *E. coli *and *S. typhi*, and data from these two species are taken to form one clade for PhyloScan. Each of the remaining species formed a separate clade of unaligned sequence data, since these species do not exhibit sequence identity with *E. coli *or with each other [26].

Generally, we would combine sequences into a single clade if their pairwise phylogenetic distances were comparable to that between *E. coli *and *S. typhi*, or nearer.

#### Constructing multiple alignments

With only two closely related species in our set, we chose the Smith-Waterman [46] pairwise, gapped local alignment algorithm (implemented as BestFit in the Wisconsin Package Version 10.3, Accelrys Inc., San Diego, CA) to align their orthologous intergenic regions, using default parameters (match = 10.000; mismatch = -9.000; gap creation penalty = 50; gap extension penalty = 3). The alignment of *E. coli *and *S. typhi *orthologous upstream intergenic sequences resulted in 1662 unique aligned sequence pairs. The upstream intergenic sequences for an additional 836 *E. coli *genes that did not have orthologs in *S. typhi *remained. The combination of these two datasets (1662 + 836 = 2498) does not equal the above number of *E. coli *intergenic regions of interest (2379 sequences), due to the complication of divergently transcribed genes. Specifically, we observed that for some divergently transcribed genes in *E. coli*, the orthologous genes in *S. typhi *are not syntenic, thus *S. typhi *provided two separate intergenic regions for alignment to a single intergenic region of *E. coli*.

To perform the real-data tests, three databases representing the reference species clade were generated for scanning: (1) a database containing the 2379 *E. coli *intergenic regions of interest, (2) a database containing only *E. coli *data ("*E. coli *reduced"), where 1662 *E. coli *intergenic regions have been reduced in sequence space by alignment with *S. typhi *orthologous data plus an additional 836 *E. coli *sequences for which there was no orthologous *S. typhi *data, and (3) a database containing 1662 *E. coli-S. typhi *aligned orthologous intergenic regions plus an additional 836 *E. coli *sequences for which there was no orthologous *S. typhi *data.

#### Producing a phylogenetic tree

We constructed the phylogenetic tree for the more complicated, synthetic sequence data set using 16S rRNA gene data via MUSCLE [30] and PHYLIP [31], scaling tree branch lengths up by a factor of 13.5, as described above – see Synthetic Sequence Data in the Results section. A tree constructed in this manner is not definitive but should be sufficient for use with PhyloScan.

#### Obtaining binding site motif models

**Figure 7:**
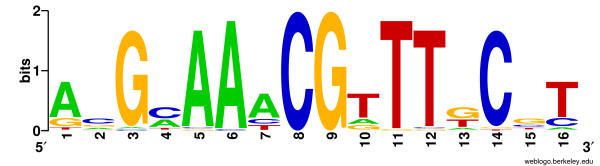
**PurR Binding Site Motif**. Shown is the PurR motif used to scan for PurR binding sites. The binding site equilibria were calculated from sequence data aligned by the Gibbs Recursive Sampler [49], and were plotted using publicly available software [27].

*E. coli *Crp and PurR binding sites that have been experimentally identified by DNase I footprinting were extracted from the literature and available databases, RegulonDB [47] and DPInteract [48]. The 87 Crp sites (from 65 *E. coli *intergenic regions) and 22 PurR sites (from 20 *E. coli *intergenic regions), were aligned using the Gibbs Recursive Sampler [49] specifying palindromic models (total width of 16–24 bp), to generate a PurR motif (Figure 7) and a Crp motif (Figure 1). These figures show both the nucleotide equilibrium and the information content for each position of the motif [9].

### Generation of the weak synthetic sequence data

To test the sensitivity and specificity of PhyloScan when seeking binding sites that are weaker than *E. coli *Crp binding sites, we generated "1/2-strength" and "1/3-strength" Crp sites. The 1/2-strength Crp motif was designed to have an average information content per column that is half the average information content of the full-strength Crp motif; we did this by raising each probability of a nucleotide to its 0.637^th ^power, with subsequent scaling so that the probabilities of the four nucleotides for any motif column sum to 1.0. Likewise, the 1/3-strength Crp sites were generated from a 1/3-strength Crp motif to give one-third the average information content, using an exponent of 0.507. See Figure 1 and its legend for more information.

## Competing interests

The authors declare that they have no competing interests.

## Authors' contributions

CSC implemented the Perl portions of the algorithm, managed the input data, and collected the output data. LAN designed and implemented the PhyloScan algorithm in C++. LAM chose the specific transcription factors to address, identified relevant input data and interpreted the algorithm output. CEL conceived the study, and participated in its design and coordination. All authors contributed to and approved the final manuscript.

## Supplementary Material

Additional file 1 **Additional Information**. This file includes legends for Supplementary Tables 2–4, which are included as additional files (see below). It includes samples of calculations described in Methods. Click here for file

Additional file 3 **Supplementary Table 3**. This table lists the sites and the *q*-values for each of the Crp binding site prediction experiments in Table 1 of the text. Click here for file

Additional file 4 **Supplementary Table 4**. This table lists the sites and the *q*-values for each of the PurR binding site prediction experiments in Table 1 of the text. Click here for file

Additional file 2 **Supplementary Table 2**. This table lists the orthologs and the orthologous intergenic regions used in this study. Click here for file
