# Supplementary material for: PhyloScan: identification of transcription factor binding sites using cross-species evidence
Source: Algorithms Mol Biol. 2007 Jan 23;2:1. doi: 10.1186/1748-7188-2-1 (PMC1794230; doi:10.1186/1748-7188-2-1)
Supplement: Additional file 1 — Additional Information. This file includes legends for Supplementary Tables 2–4, which are included as additional files (see below). It includes samples of calculations described in Methods. [file 1748-7188-2-1-S1.doc]

**SUPPLEMENTARY DATA**

**Supplementary Table 2 caption.** The Excel data table lists the orthologs and the orthologous intergenic regions used in this study. Species abbreviations are: *E. coli* (ECOL), *S. typhi* (STYP), *Y. pestis* (YPES), *H. influenzae* (HINF), *V. cholerae* (VCHO), *S. oneidensis* (SONE), *P. aeruginosa* (PAER). For each *E. coli* gene, the common gene name and the upstream intergenic genomic coordinates are listed, followed by paired columns containing the orthologous information for each of the additional species, *i.e.*, the name of the orthologous gene in each species and the corresponding upstream intergenic genomic coordinates. In cases where an ortholog of a particular *E. coli* gene was not present in a species, these columns contain a “-“. There were cases in which an orthologous gene was identified, but for which the upstream intergenic region was < 20 bp, in these cases the gene name is listed but no genome coordinates are provided (*e.g*.: *E. coli* gene *acrB* has an ortholog in *H. influenzae*, HI0895, but this gene’s upstream intergenic region was < 20 bp).

**Supplementary Table 3 caption.** Excel sheets of the sites and the *q*-values for each of the Crp binding site prediction experiments in Table 1 of the text. The first sheet lists the Crp binding sites identified in the database of 2379 *E. coli* intergenic regions (described above) when using a product multinomial model (*q*-values in column E) and when combining evidence from the additional, independent species (*q*-values in column F); in this database, the number of sites evaluated for the Crp model was 390,054. The second sheet lists the Crp binding sites identified in the database of “reduced” *E. coli* intergenic regions (described above) when using a product multinomial model (*q*-values in column E) and when combining evidence from the additional, independent species (*q*-values in column F); in this database, the number of sites evaluated for the Crp model was 289,607. The third sheet lists the Crp binding sites identified in the database of aligned *E. coli* - *S. typhi* intergenic regions (described above) when using a product phylogeny model (*q*-values in column E) and when combining evidence from the additional, independent species (*q*-values in column F); in this database, the number of sites evaluated for the Crp model was 270,205. For Crp predictions, the independent species data used were from *Y. pestis*, *H. influenzae*, *V. cholerae*, *S. oneidensis*, and *P. aeruginosa*.

**Supplementary Table 4 caption**. Excel sheets of the sites and the *q*-values for each of the PurR binding site prediction experiments in Table 1 of the text. The first sheet lists the PurR binding sites identified in the database of 2379 *E. coli* intergenic regions (described above) when using a product multinomial model (*q*-values in column E) and when combining evidence from the additional, independent species (*q*-values in column F); in this database, the number of sites evaluated for the PurR model was 404,328. The second sheet lists the PurR binding sites identified in the database of “reduced” *E. coli* intergenic regions (described above) when using a product multinomial model (*q*-values in column E) and when combining evidence from the additional, independent species (*q*-values in column F); in this database, the number of sites evaluated for the PurR model was 308,410. The third sheet lists the PurR binding sites identified in the database of aligned *E. coli* - *S. typhi* intergenic regions (described above) when using a product phylogeny model (*q*-values in column E) and when combining evidence from the additional, independent species (*q*-values in column F); in this database, the number of sites evaluated for the PurR model was 292,084. For PurR predictions, the independent species data used were from *Y. pestis*, *H. influenzae*, and *V. cholerae*.

**Example of PhyloScan calculations.** The following example provides an illustration of how the combined evidence *p*-value and the *q*-value are calculated for a known PurR TFBS upstream from the *E. coli* gene *guaB.* Orthologous intergenic regions for *guaB* were identified for all of the additional species used in this study (*S. typhi, Y. pestis, V. cholerae, H. influenzae, S. oneidensis* and *P. aeruginosa*); however, because a PurR ortholog was not detected in either *S. oneidensis* or *P. aeruginosa,* these species were not used when we combined evidence predicting PurR TFBSs.

*E. coli –* *S. typhi p*-value = 1.697 x 10^-5^

*Y. pestis* *p*-value = 2.551 x 10^-4^

*V. cholerae* *p*-value = 3.119 x 10^-3^

*H. influenzae* *p*-value = 0.8058

$$p=\prod_{i=1}^{n} P_{i}=(1.697\times10^{-5})(2.551\times10^{-4})(3.119\times10^{-3})(0.8058)=1.0880 \times10^{-11}$$

$$\begin{matrix} \sum_{i=0}^{n-1} \frac{\left(-\ln\frac{p}{0.05}\right)^{i}}{i!}= \\ \frac{\left(-\ln\left(\frac{1.088\times10^{-11}}{0.05}\right)\right)^{0}}{0!}+\frac{\left(-\ln\left(\frac{1.088\times10^{-11}}{0.05}\right)\right)^{1}}{1!}+\frac{\left(-\ln\left(\frac{1.088\times10^{-11}}{0.05}\right)\right)^{2}}{2!}+\frac{\left(-\ln\left(\frac{1.088\times10^{-11}}{0.05}\right)\right)^{3}}{3!}= \\ \\ 1+\left(2.2248\times10^{1}\right)+\left(2.4749\times10^{2}\right)+\left(1.8355\times10^{3}\right)=2.1062\times10^{3} \end{matrix}$$

The combined evidence *p*-value is $F\left(p\right)=(1.0880\times10^{-11})(2.1062\times10^{3})=2.292\times10^{-8}$, where the value $2.1062\times10^{3}$ is the “correction” factor, which takes into account all of the different combinations of *p*-values that could have resulted in the product $1.0880\times10^{-11}$. The *q*-value is then calculated, considering that there were 2415 sequences examined (after alignment with *S. typhi* orthologous data, only 2415 of the total 2498 *E. coli* intergenic regions were long enough to accommodate a PurR site), and the *guaB* intergenic region had the 8^th^ best combined *p*-value:

$$q=\frac{(2.292\times10^{-8})(2415)}{8}=6.918\times10^{-6}$$
